# Arhgef15 Promotes Retinal Angiogenesis by Mediating VEGF-Induced Cdc42 Activation and Potentiating RhoJ Inactivation in Endothelial Cells

**DOI:** 10.1371/journal.pone.0045858

**Published:** 2012-09-21

**Authors:** Sentaro Kusuhara, Yoko Fukushima, Shigetomo Fukuhara, Lars Martin Jakt, Mitsuhiro Okada, Yuri Shimizu, Masayuki Hata, Kohji Nishida, Akira Negi, Masanori Hirashima, Naoki Mochizuki, Shin-Ichi Nishikawa, Akiyoshi Uemura

**Affiliations:** 1 Laboratory for Stem Cell Biology, RIKEN Center for Developmental Biology, Kobe, Japan; 2 Division of Ophthalmology, Department of Surgery, Kobe University Graduate School of Medicine, Kobe, Japan; 3 Division of Vascular Biology, Department of Physiology and Cell Biology, Kobe University Graduate School of Medicine, Kobe, Japan; 4 Department of Ophthalmology, Osaka University Medical School, Osaka, Japan; 5 Department of Cell Biology, National Cerebral and Cardiovascular Center Research Institute, Osaka, Japan; University of Regensburg, Germany

## Abstract

**Background:**

Drugs inhibiting vascular endothelial growth factor (VEGF) signaling are globally administered to suppress deregulated angiogenesis in a variety of eye diseases. However, anti-VEGF therapy potentially affects the normal functions of retinal neurons and glias which constitutively express VEGF receptor 2. Thus, it is desirable to identify novel drug targets which are exclusively expressed in endothelial cells (ECs). Here we attempted to identify an EC-specific Rho guanine nucleotide exchange factor (GEF) and evaluate its role in retinal angiogenesis.

**Methodology/Principal Findings:**

By exploiting fluorescence-activated cell sorting and microarray analyses in conjunction with in silico bioinformatics analyses, we comprehensively identified endothelial genes in angiogenic retinal vessels of postnatal mice. Of 9 RhoGEFs which were highly expressed in retinal ECs, we show that Arhgef15 acted as an EC-specific GEF to mediate VEGF-induced Cdc42 activation and potentiated RhoJ inactivation, thereby promoting actin polymerization and cell motility. Disruption of the Arhgef15 gene led to delayed extension of vascular networks and subsequent reduction of total vessel areas in postnatal mouse retinas.

**Conclusions/Significance:**

Our study provides information useful to the development of new means of selectively manipulating angiogenesis without affecting homeostasis in un-targeted tissues; not only in eyes but also in various disease settings such as cancer.

## Introduction

Angiogenesis is the process of the formation of vascular networks characterized by sprouting, branching, and regression of new blood vessels [1]. Because vascular endothelial growth factor (VEGF, also known as VEGF-A) plays predominant roles in this complex process by promoting proliferation, migration, and survival of endothelial cells (ECs), drugs inhibiting VEGF signaling have been globally administered to suppress deregulated angiogenesis in a variety of eye diseases, including age-related macular degeneration [2], [3] and retinopathy of prematurity [4]. However, adverse effects of VEGF deprivation have been indicated in the normal functions of retinal neurons and Müller glias which constitutively express VEGF receptor 2 (VEGFR2) [5]–[7]. Thus, it is desirable to develop an alternative modality which can selectively target abnormal vessels, without affecting homeostasis in neural tissues.

The small GTPase Cdc42, which cycles between an active, GTP-bound state and an inactive, GDP-bound state, facilitates actin polymerization in various types of cells and is critically involved in diverse cell processes, such as cell motility [8]. In ECs, Cdc42 is activated by binding of VEGF to VEGFR2, whereas binding of semaphorin 3E (Sema3E) to PlexinD1 receptor inactivates Cdc42 [9], [10]. By contrast, RhoJ, which displays 55% homology to Cdc42 in its amino acid sequences, is inactivated by VEGF and activated by Sema3E in ECs [10]. Intriguingly, while RhoJ binds to effector proteins of Cdc42 such as p21-activated kinase (PAK) and neural Wiskott-Aldrich syndrome protein [11], [12], RhoJ induces actin depolymerization in ECs [10]. Thus, the inverse regulation of the activation status of Cdc42 and RhoJ downstream of VEGF-VEGFR2 and Sema3E-PlexinD1 signals are the pivotal intracellular events to mediate the cytoskeletal reorganization in ECs. Because small molecule inhibitors targeting guanine nucleotide exchange factors (GEFs) are expected to have therapeutic value [13], [14], an endothelial GEF which activates Cdc42 or RhoJ would be a promising target for novel anti-angiogenic therapies.

Here, by utilizing fluorescence-activated cell sorting (FACS) and microarray transcriptome profiling in conjunction with *in silico* bioinformatics analyses, we show that Arhgef15 (also known as Vsm-RhoGEF [15] and Ephexin5 [16]) acts as an EC-specific GEF to mediate VEGF-induced Cdc42 activation and further potentiates RhoJ inactivation, thereby promoting actin polymerization. Inactivation of the *Arhgef15* gene resulted in retardation of retinal vascular growth, indicating Arhgef15 as a potential drug target.

## Results

### Transcriptome Analysis in ECs of Living Mouse Retinas

**Figure 1:**
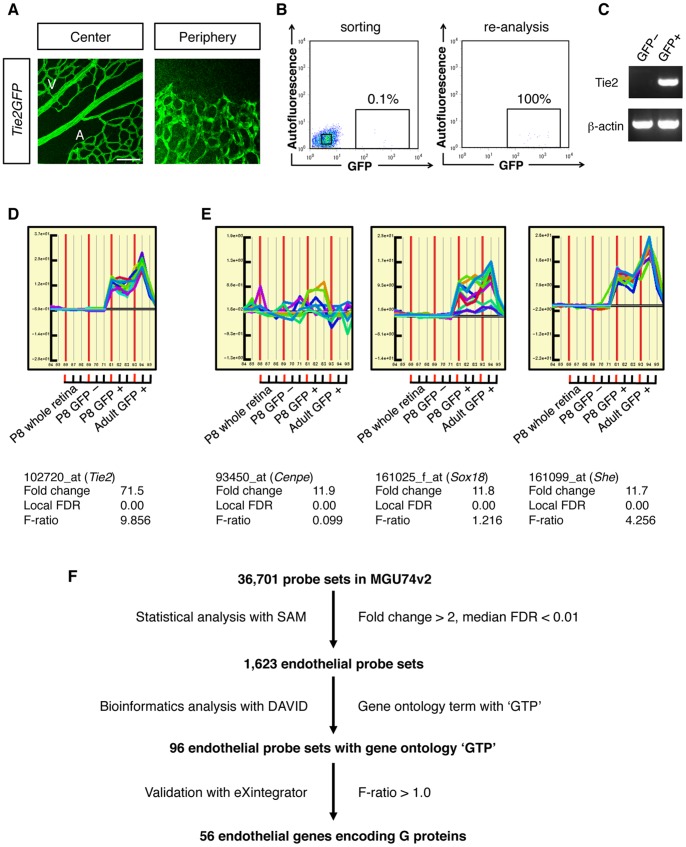
FACS and microarray analysis in mouse retinas. (A) Whole-mount retinal immunohistochemistry (IHC) for GFP in P6 *Tie2GFP* Tg mouse. GFP is uniformly expressed in ECs over the developing retinal vasculature. A, artery; V, vein. Scale bar: 100 µm. (B) FACS purification of GFP-positive ECs from retinas of P8 homozygous *Tie2GFP* Tg mouse. Re-analysis of sorted GFP-positive cells showed 100% purity. (C) RT-PCR from total RNA of GFP-negative and GFP-positive cells sorted from P8 *Tie2GFP* Tg retinas. (D) *In silico* validation of the microarray data by the eXintegrator system. The signal intensities of individual member probes of the *Tie2* probe set are displayed as colored lines. *Tie2* probe set probes are uniformly and exclusively up-regulated in all GFP-positive samples. The *x* axis represents samples (with 3 replicates of each cell type) and the *y* axis signal intensity. Vertical red lines indicate borders between sample groups. (E) Representative images showing the discrepancy between statistics values and probe-pair signals. Despite the equivalent fold change and FDR values, the eXintegrator analysis for 93450_at (*Centromere protein e*, *Cenpe*), 161025_f_at (*Sry-box containing gene 18*, *Sox18*), and 161099_at (*Src homology 2 domain-containing transforming protein e*, *She*) indicates a high variability in internal correlation of individual probes. This can also be inferred from f-ratios calculated as the ratio of variance between sample means to variance within probe set member probes after individual z-score normalization of member probes across the sample series. (F) A scheme for the identification of endothelial G proteins.

In order to identify RhoGEFs which are highly expressed in retinal ECs, we performed transcriptome analyses in mouse retinas, in which new blood vessels begin to grow radially from the optic disc shortly after birth, and subsequently form a network in the most superficial layer [17], [18]. Until the growing blood vessels reach the retinal periphery around postnatal day 9 (P9), new blood vessels continuously sprout at the leading fronts, whereas perpendicular vessels sprout from the preformed veins and capillaries around P8 to form the deep and intermediate vascular layers. To comprehensively analyze endothelial gene expression at these angiogenic stages, we dissociated retinas of P8 *Tie2GFP* transgenic (Tg) mice into single cells, and purified GFP-positive ECs by FACS (Figure 1A, B, and Figure S1A). Because retinas of hemizygous *Tie2GFP* Tg mice showed incomplete separation of GFP-positive and GFP-negative cell fractions (Figure S1B), we employed homozygous *Tie2GFP* Tg mice in all following experiments. In retinas of P8 *Tie2GFP* Tg mice, only 0.1% of the cell population represented GFP-positive cells. Accordingly, we obtained less than 500 GFP-positive cells per retina. However, re-analysis of sorted GFP-positive cells ensured nearly 100% purity (Figure 1B), which was further confirmed by the expression of the *Tie2* transcript exclusively in the GFP-positive cell fraction (Figure 1C). We then performed microarray analysis on these cells. Using the Significance Analysis of Microarrays (SAM) algorithm [19] with a fold change >2 and a median false discovery rate (FDR) <0.01, we identified 1,623 probe sets out of 36,701 probe sets as having higher signals in the GFP-positive cells than in the GFP-negative cells in P8 retinas (Dataset S1).

To identify endothelial genes encoding guanine nucleotide binding (G) proteins, we used the online NIH-DAVID software to extract probe sets whose gene ontology terms included ‘GTP’, such as ‘GTP binding’, ‘GTPase activity’, ‘GTPase activator activity’, ‘small GTPase regulator activity’, and ‘small GTPase mediated signal transduction’. Of the 1,623 endothelial probe sets, 96 probe sets were related to the ‘GTP’ gene ontology terms (Dataset S2).

To ensure the integrity of microarray data, the exclusion of false-positive and false-negative errors is inevitably required [20]. In the present study, we performed *in silico* validation of the microarray data using the eXintegrator system, which displays the individual probe intensities in distinct probe sets across multiple samples [21]. We inspected microarray data sets obtained from entire P8 retinas (un-sorted), GFP-negative and GFP-positive P8 retinal cells, as well as GFP-positive cells from adult retinas (*n* = 3 for each population, Figure 1D). Importantly, microarray data with similar fold-change and FDR estimates showed variable degrees of internal correlation among probe-set member probes (Figure 1E). Therefore, we evaluated the internal probe-set consistency by visually inspecting the identified probe sets (Figure S2) and extracted probe sets having f-ratios (variance between sample means to variance within member probes) higher than 1.0 using the eXintegrator system (Figure 1F). Thus we determined 56 endothelial genes encoding G proteins in postnatal mouse retinas (Table S1).

### Arhgef15 Mediates VEGF-induced Cdc42 Activation and Potentiates RhoJ Inactivation

**Figure 2:**
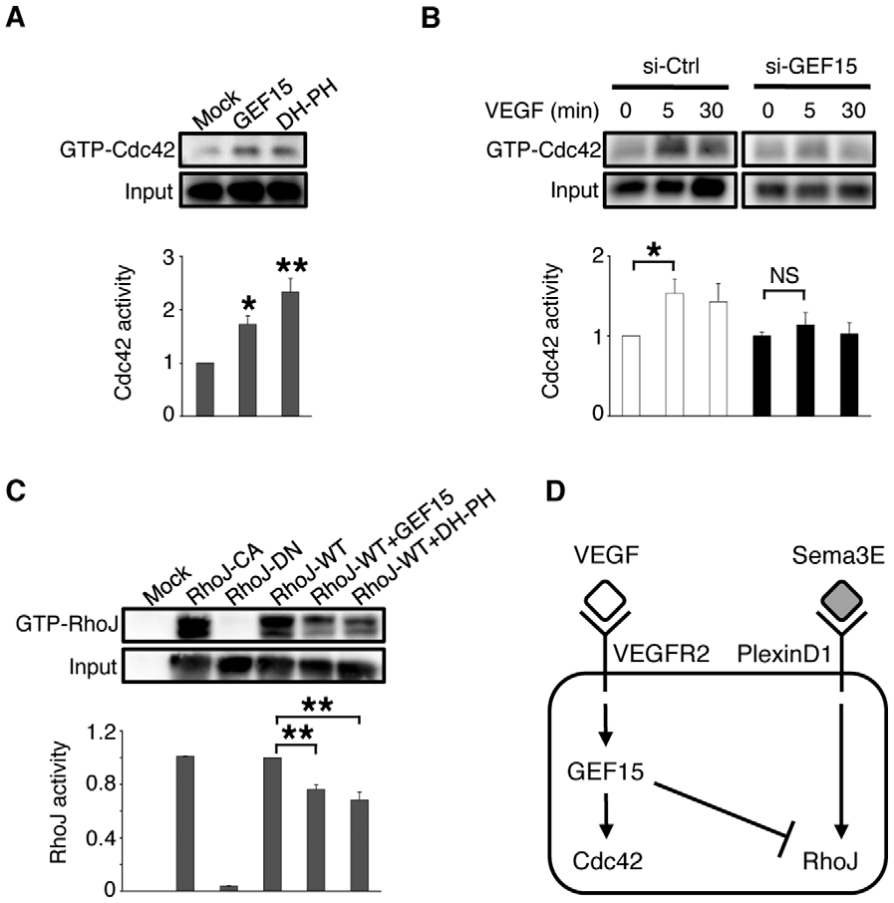
Arhgef15 mediates VEGF-induced Cdc42 activation and potentiates RhoJ inactivation. (A) Ectopic expression of full-length Arhgef15 (GEF15) and its DH-PH truncated proteins activated endogenous Cdc42 in 293T cells. (B) VEGF-induced Cdc42 activation at 5 min was abolished by Arhgef15 knockdown in HUVECs. (C) Ectopic expression of GEF15 and its DH-PH proteins inactivated co-transfected RhoJ-WT in 293T cells. CA, constitutively-active; DN, dominant negative. In A–C, representative immunoblotting images of 3 independent experiments are shown. Error bars represent SEM; *P<0.05, **P<0.01. (D) A scheme xrepresenting signal transduction in ECs.

In the list of endothelial G proteins, 9 RhoGEFs were identified, including *Fgd5* (164116_at), *Dock9* (104714_at), *Arhgef15* (113016_at), *LOC670024* (115197_at), *Net1* (94223_at), *Spata13* (100958_at), *Dock1* (109494_at), *Arhgef7* (98434_at), and *Arhgef12* (115082_at). Based on internal probe-set probe co-variance as displayed by the eXintegrator system, and retinal whole-mount *in situ* hybridization (ISH, Figure S3A), we focused on Arhgef15, which contains Dbl homology (DH) and pleckstrin homology (PH) domains required for RhoGEF activity and membrane binding, respectively [22]. In 293T cells, ectopically expressed Arhgef15 as well as its truncated DH-PH proteins induced activation of endogenous Cdc42 (Figure 2A). Furthermore, VEGF-induced activation of Cdc42 was abrogated by Arhgef15 knockdown in human umbilical vascular ECs (HUVECs, Figure 2B and Figure S4). By contrast, ectopic Arhgef15 and its DH-PH form inactivated co-transfected RhoJ in 293T cells (Figure 2C). These results together indicate that Arhgef15 mediates VEGF-induced Cdc42 activation and further potentiates RhoJ inactivation in ECs (Figure 2D).

### Arhgef15 Facilitates Actin Polymerization and Cell Motility in ECs

**Figure 3:**
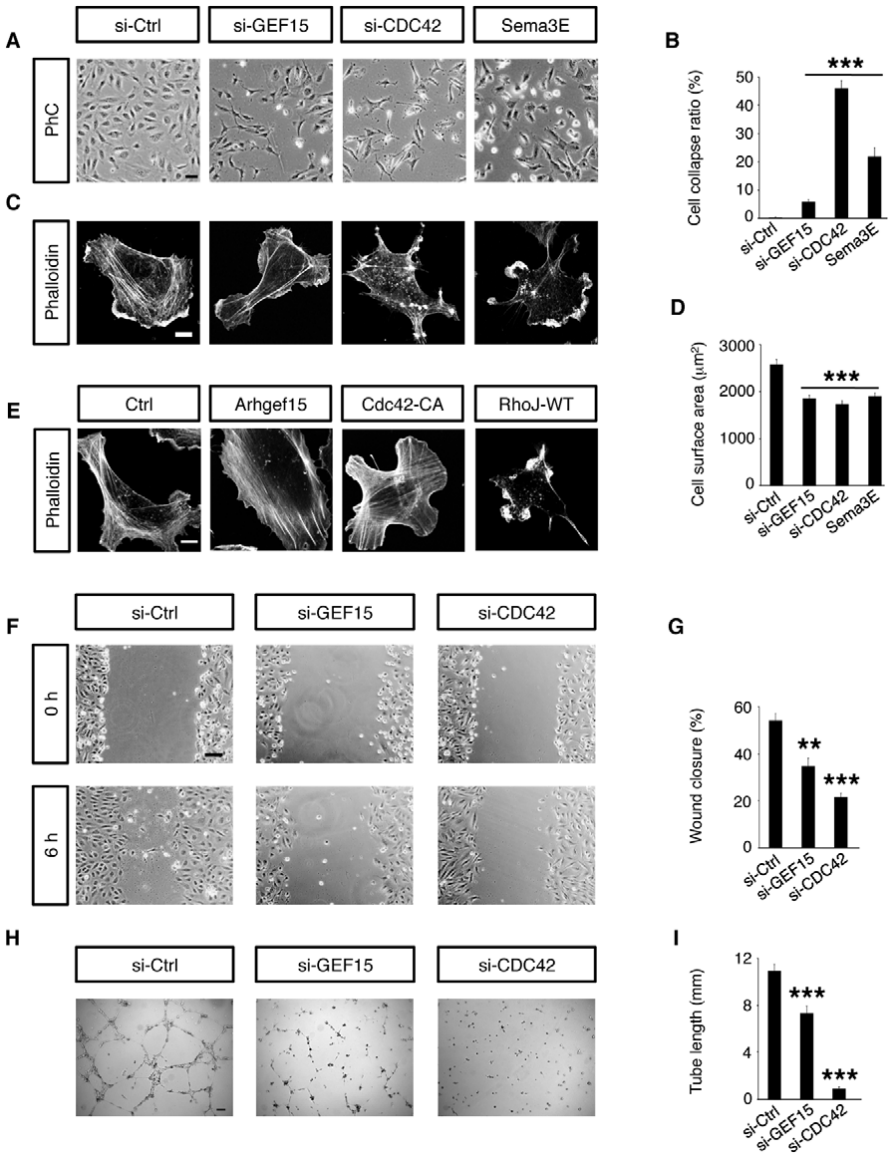
Arhgef15 facilitates actin polymerization and cell motility in ECs. (A) Phase-contrast (PhC) microscopy in cultured HUVECs at 72 h after siRNA transfection or 30 min after Sema3E stimulation. (B) The ratio of collapsing cells in cultured HUVECs. All experiments were repeated at least four times. (C) Confocal microscopy for phalloidin in cultured HUVECs. Note the actin depolymerization after transfection of siRNAs for Arhgef15 and Cdc42, and after Sema3E stimulation. (D) Quantification of the cell surface area in cultured HUVECs. The number of cells analyzed were: si-Ctrl, 151; si-GEF15, 170; si-CDC42, 151; Sema3E, 168. (E) Confocal microscopy for phalloidin in cultured HUVECs. Note the thickening of actin fibers at 24 h after transfection of plasmid vectors expressing Arhgef15 and Cdc42-CA, in contrast to the disruption of actin fibers by RhoJ-WT overexpression. (F) Scratch-wound assay in HUVECs transfected with siRNAs. Note the reduced EC motility by knockdown of Arhgef15 and Cdc42. (G) Quantification of the wound closure. *n* = 3 per group. (H) Tube formation assay in HUVECs transfected with siRNAs. Note the inhibition of capillary-like network formation by knockdown of Arhgef15 and Cdc42. (I) Quantification of the total tube length. *n* = 7 per group. Scale bar: 50 µm (A); 10 µm (C and E); 100 µm (F and H). Error bars represent SEM; **P<0.01, ***P<0.001.

To assess the roles of Arhgef15 in ECs, we transfected siRNA for Arhgef15 in cultured HUVECs. Arhgef15 knockdown did not affect the cell proliferation and apoptosis (Figure S5), but induced depolymerization of actin filaments and a 28% decrease in cell surface areas, leading to cell collapse in 5.9% of siRNA-treated cells (Figure 3A–D). Similar effects were observed by Cdc42 knockdown or Sema3E stimulation, but with higher rates of cell collapse (Figure 3A–D). In addition to HUVECs, actin depolymerization by Arhgef15 knockdown was also induced in cultured human retinal microvascular ECs (HRECs, Figure S6). By contrast, a small population of Arhgef15-overexpressing HUVECs formed thickened actin fibers, which was more frequently observed in HUVECs overexpressing constitutively-active Cdc42 (Figure 3E). Together with the disruption of actin fibers in RhoJ-overexpressing HUVECs (Figure 3E), these results indicate that Arhgef15 facilitates actin polymerization by activating Cdc42 and inactivating RhoJ in ECs.

Because reorganization of actin cytoskeletons is prerequisite for cell motility [23], we further performed scratch-wound assay and tube formation assay after siRNA transfection. Arhgef15 knockdown significantly reduced EC migration (Figure 3F, G) and capillary-like network formation (Figure 3H, I), which was more pronounced by Cdc42 knockdown. Thus, Arhgef15 plays a pivotal role for the angiogenic activities in ECs.

### Arhgef15 Promotes Extension of Retinal Vascular Networks

**Figure 4:**
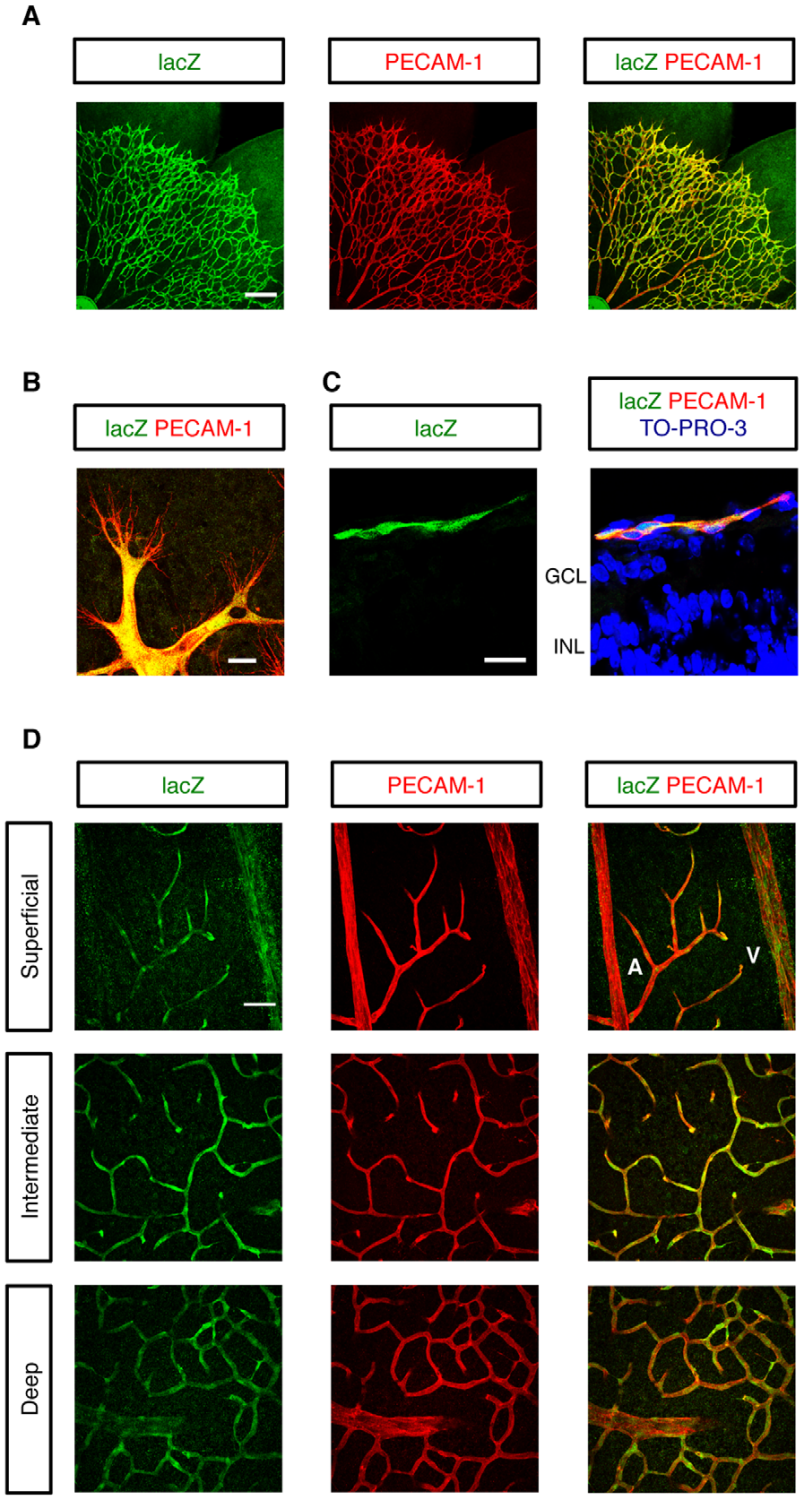
EC-specific expression of Arhgef15 in mouse retinas. (A–D) IHC for lacZ and PECAM-1 in whole-mount retinas (A, B, and D) and retinal cryo-sections (C) of *Arhgef15* ^lacZ/lacZ^ mice at P5 (A–C) and P17 (D). Note the endothelial specificity of Arhgef15 expression in mouse retinas at early and late postnatal stages. At P17, endothelial expression of Arhgef15 in the superficial vascular layer was diminished in arteries but was maintained in veins. A, artery; V, vein. Scale bar: 200 µm (A); 20 µm (B and C); 50 µm (D).

**Figure 5:**
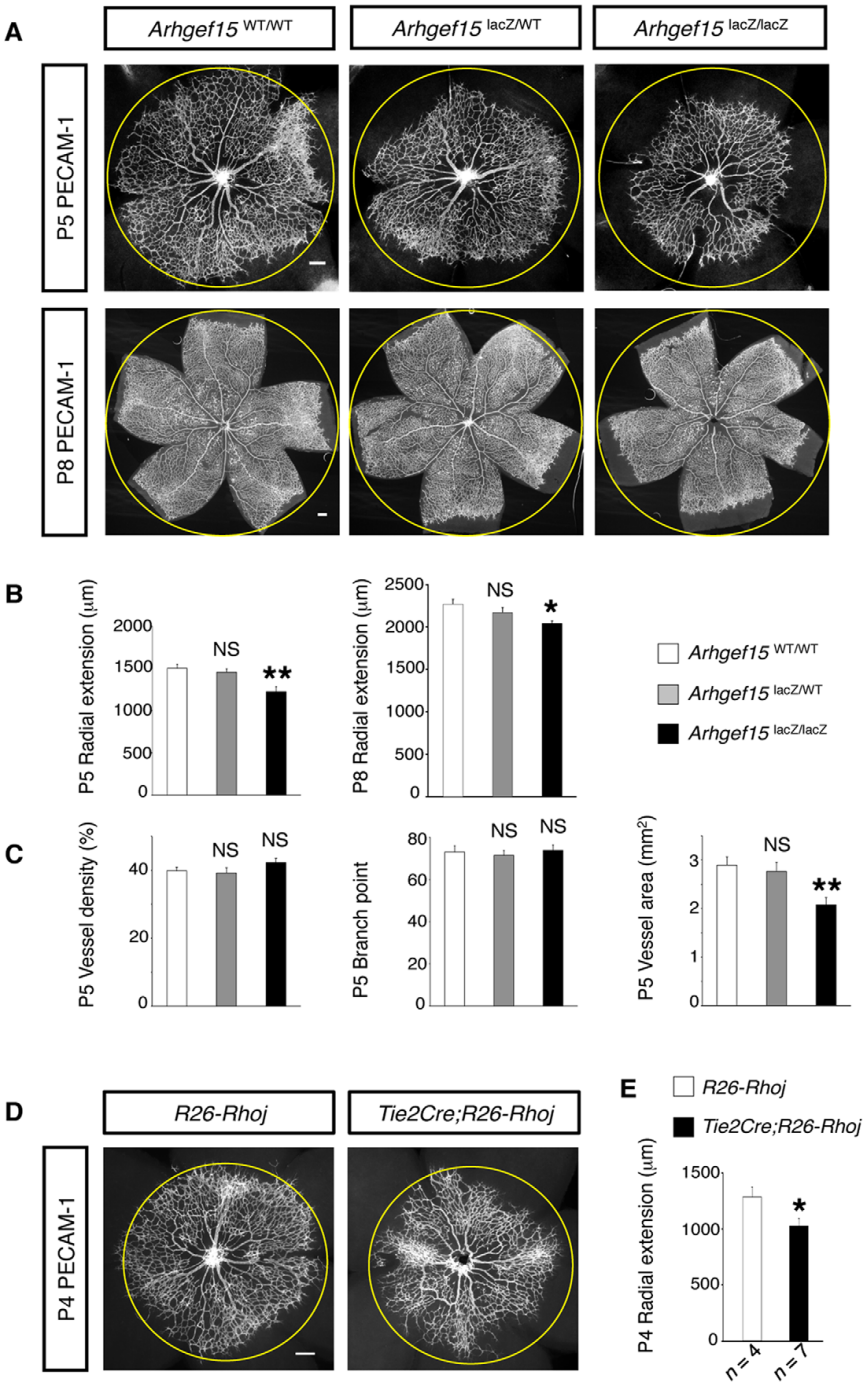
Retardation of retinal vascular growth in *Arhgef15*-KO mouse. (A) Whole-mount IHC for PECAM-1 in P5 and P8 retinas. Circles represent the vascular margin of the *Arhgef*15^WT/WT^ retinas. (B) Quantification of the radius of the retinal vascular networks (P5, *n* = 6; P8, *n* = 4). (C) Morphometric analyses of vascular networks in P5 retinas (*n* = 6 per group). (D) Whole-mount IHC for PECAM-1 in P4 retinas. Circles represent the vascular margin of the *R26-Rhoj* retina. (E) Quantification of the radius of the retinal vascular networks at P4. Scale bar: 200 µm. Error bars represent SEM; *P<0.05, **P<0.01.

To evaluate the contributions of Arhgef15 to retinal angiogenesis, we generated *Arhgef15* knock-out (KO) mice, in which endogenous *Arhgef15* expression can be monitored by lacZ expression (Figure S7). In agreement with our ISH experiments, lacZ was expressed in ECs over the developing retinal vasculature in *Arhgef15*-KO mouse (Figure 4A). Higher magnification images further demonstrated lacZ expression in ECs both at the tips and stalks of sprouting vessels (Figure 4B and Figure S8A). Moreover, lacZ expression was undetectable in neurons, astrocytes, and vascular smooth muscle cells (vSMCs), ensuring the endothelial specificity of Arhgef15 expression in mouse retinas (Figure 4C and Figure S8B–D). At later postnatal stages, while Arhgef15 was uniformly expressed in ECs of deeper vascular layers, its expression was faint in arterial ECs of the superficial vasculature. (Figure 4D). Although pups homozygous for the *Arhgef15* null alleles were viable without any gross abnormalities, the radial extension of the retinal vascular network was retarded by 18.5% at P5 and by 9.9% at P8, compared to wild-type (WT) mice (Figure 5A, B). Because the growing blood vessels in the *Arhgef15*-KO mice reached the retinal periphery at P10 and formed deeper vascular layers as in WT mice (Figure S9), the retinal vascular retardation at early postnatal stages is likely to be ascribable to the impaired EC motility due to the lack of Arhgef15. In P5 *Arhgef15*-KO retinas, while the vessel density and the number of vessel branch points were unaffected, the delayed vascular extension resulted in 28.3% reduction of the total vessel area (Figure 5C). Interestingly, the degree of vascular growth retardation in *Arhgef15*-KO retinas was equivalent to that caused by endothelial RhoJ overexpression (Figure 5D, E), indicating that Arhgef15 promotes extension of retinal vascular networks, at least in part, by inactivating RhoJ in ECs.

## Discussion

In order to identify novel target molecules for the treatment of neovascular eye diseases, we performed comprehensive transcriptome analyses in postnatal mouse retinas, because a considerable number, if not all, of endothelial genes in developing retinal vessels are also expected to be expressed in pathological angiogenesis. To date, a series of high-throughput analyses exploiting cultured or tissue-derived ECs have shown that endothelial gene expression fluctuates depending on microenvironments [24]–[26]. This notion prompted us to isolate retinal ECs from *Tie2GFP* Tg mice, which can reduce alterations in gene expression by omitting time-consuming antibody labeling steps. Indeed, the bioinformatics analysis of our microarray data using the NIH-DAVID software demonstrated that most of the gene ontology terms (level 3 biological processes with *p*-value <10^−5^) enriched in the 1,623 endothelial genes were associated with dynamic cell behavior such as “cell motility”, “cell migration”, and “angiogenesis” (Table S2). However, we should be aware of drawbacks inherent in our FACS and microarray analyses. Firstly, the expression levels of short-life genes, such as those up-regulated under hypoxia, may be affected during the sample preparation. Secondly, endothelial genes expressed at limited sites, such as sprouting vascular tips [27], [28], might be masked by the lack of expression in the majority of ECs. Thirdly, the relative comparison of gene expression levels between ECs and non-ECs does not necessarily extract EC-specific genes. To overcome these obstacles, the exact expression pattern of particular genes should be determined by highly sensitive procedures, such as the use of reporter mouse strains.

While 80 RhoGEFs comprising the Dbl family and the Dock family have been identified [22], we successfully discovered that Arhgef15 acts as an EC-specific GEF to activate Cdc42 downstream of VEGF signaling. Although endothelial Arhgef15 expression was also detected in embryonic vasculature (Figure S3B), previous literature reported Arhgef15 expression in vSMCs [15] and in neurons [16]. Thus, the Arhgef15 expression should further be examined in various tissues during development and in adults. In addition to Cdc42 activation, Arhgef15 potentiated RhoJ inactivation, which may underlie VEGF-induced RhoJ inactivation [10]. In this process, a distinct GTPase activating protein [13] downstream of Arhgef15 or Cdc42, may directly inactivate RhoJ. The full understanding of the Arhgef15-mediated signaling cascades will provide further information for the discovery of novel drug targets.

Based on the impaired vascular growth in *Arhgef15*-KO retinas, we propose that Arhgef15 is a potential molecular target for selectively manipulating angiogenesis without affecting tissue homeostasis. Given the lower collapse rate of HUVECs induced by Arhgef15 knockdown than by Cdc42 knockdown, it seems likely that multiple GEFs, such as Fgd5 [29], are involved in Cdc42 activation in ECs. It is also possible that Arhgef15 modulates the activity of alternative Rho small GTPases, such as RhoA [15], [16]. Therefore, enhanced anti-angiogenic effects may be achieved by targeting multiple endothelial G proteins. For this purpose, our transcriptome profiling is a useful source of information to identify novel target molecules for the treatment of neovascular eye diseases, as well as diseases such as cancer characterized by deregulated angiogenesis.

## Materials and Methods

### Ethics Statement

All experimental protocols adhered to the Association for Research in Vision and Ophthalmology (ARVO) Statement for the Use of Animals in Ophthalmic and Vision Research, and were reviewed and approved by the Institutional Animal Care and Use Committee of RIKEN CDB (permit number H15-9).

### Mice

*Tie2GFP* Tg mice [30] with an FVB/N background (stock number 003658) were purchased from Jackson Laboratory. For the generation of *Arhgef15*-KO mice, a 9.9 kb fragment containing 5′ untranslated sequences of exons 1 and 2, and a 1.2 kb fragment containing exons 5 and 6 were synthesized by PCR using a C57BL/6-derived BAC clone RP23-396M19 (BACPAC Resource Center) as a template. A targeting vector was constructed by inserting the 5′ and 3′ arms on each side of a *lox71-LacZ-pA-PGK-Neo-lox2272-pA-FRT* cassette [31] in a *pKO Scrambler NTKV-1906* vector (Stratagene). The *MC1-TK-pA* cassette was placed downstream of the 3′ arm for negative selection. Gene targeting was done with TT2 ES cells [32] and confirmed by PCR and Southern blotting. Mice were generated by injection of targeted ES cells into ICR embryos, and backcrossed to C57BL/6 more than 7 times. Offsprings were genotyped by PCR using the following primers: 5′-TCTTCCTGCAGCAACGCCCC-3′ (WT 5′), 5′-CTGGGCTGGGACTCGGTGTG-3′ (WT 3′), 5′-GCTCTGATGCCGCCGTGTTCC-3′ (KO 5′), and 5′-ATAGAAGGCGATGCGCTGCG-3′ (KO 3′). Analyses of the *Arhgef15*-KO phenotypes were performed on neonates derived from in vitro fertilization using heterozygous animals. *Arhgef15*-KO animals were born at the frequency predicted for Mendelian transmission and viable without any gross abnormality. To obtain mutant pups that overexpress RhoJ in ECs, homozygous *R26-Rhoj* female mice [10] with a C57BL/6 background (Acc. No. CDB0919K: http://www.cdb.riken.jp/arg/mutant%20mice%20list.html) were mated with heterozygous *Tie2Cre* male mice (provided by R. Wang, UCSF, San Francisco, CA) [33] with a C57BL/6 background.

### FACS and Microarray

Retinas of homozygous *Tie2GFP* Tg mice were dissected in staining solution (phosphate buffered saline (PBS) containing 2 mM ethylenediaminetetraacetic acid (EDTA), 0.01% NaN_3_, 5% fetal bovine serum (FBS), 50 U/ml penicillin, and 0.05 mg/ml streptomycin), rinsed in PBS, and digested in papain solution (PBS containing 33 U/ml papain (Sigma-Aldrich), 125 U/ml DNaseI (Sigma-Aldrich), 0.4 mg/ml L-cysteine (Nacalai Tesque), and 0.5 mM EDTA) for 60 min at 37°C. Dissociated cells were treated twice with ovomucoid solution (PBS containing 2 mg/ml ovomucoid (Sigma-Aldrich), 1 mg/ml bovine serum albumin (BSA), and 125 U/ml DNaseI) for 60 min at 37°C and filtered through nylon mesh. After centrifugation, cells were incubated again in ovomucoid solution for 60 min at 37°C, rinsed twice in the staining solution, and resuspended in the staining solution containing 5 µg/ml of propidium iodide. After filtration through nylon mesh, cells were separated into GFP-positive and GFP-negative fractions by FACSAria (BD). Doublet cells were excluded using the 2-dimensional profile of forward versus side scatter. Using total RNA extracted from more than 2×10^4^ sorted cells by RNeasy Micro Kit (QIAGEN) and amplified by MessageAmpII aRNA Amplification Kit (Ambion), biotin-labeled cRNA was prepared with BioArray RNA Transcript Labeling Kit (Affymetrix) and hybridized onto the Affymetrix oligonucleotide arrays MGU74v2 as described previously [34]. Primary microarray data have been deposited in NCBI’s Gene Expression Omnibus and are accessible through GEO series accession number GSE27238 (http://www.ncbi.nlm.nih.gov/geo/query/acc.cgi?acc=GSE27238).

### Bioinfomatics Analyses

The dChip software [35] (version 1.3) was used to normalize the CEL files at probe level and compute model-based expression values using the PM only model. A two-sample comparison was performed using the SAM algorithm [19] with 800 permutations. We used a minimum two-fold change in expression and a δ-value set to give a median FDR <0.01. Gene ontology annotations and classifications were generated using the DAVID (Database for Annotation, Visualization and Integrated Discovery) software (version 6.7) from the NIH (http://david.abcc.ncifcrf.gov/). The eXintegrator system was used to manually validate probe sets identified by the above statistical method as well as to calculate f-ratios for individual probe sets (http://www.cdb.riken.jp/scb/documentation/).

### RT-PCR

Total RNA isolated from 1,000 sorted cells was used for single-step RT-PCR with OneStep RT-PCR Kit (QIAGEN), using following primers; Tie2 forward, 5′-TCTTGTGTCTGATGCCGAAAC-3′; Tie2 reverse, 5′-GCAGGTAGGAAGGACGCTTGT-3′ (282 bp); β-actin forward, 5′-TCGTGCGTGACATCAAAGAG-3′; and β-actin reverse, 5′-TGGACAGTGAGGCCAAGATG-3′ (429 bp).

### Immunohistochemistry

The procedures for preparations of retinal samples have been described elsewhere [36]. For immunohistochemistry, samples were incubated with the following primary antibodies (Abs): polyclonal rabbit anti-GFP (Life Technologies), monoclonal rat anti-PECAM-1 (clone Mec13.3; BD Biosciences), monoclonal rat anti-VE-cadherin (clone 11D4.1; BD Biosciences), polyclonal goat anti-PDGFRα (R&D Systems), Cy3-conjugated monoclonal anti-α smooth muscle actin (clone 1A4; Sigma-Aldrich), and polyclonal rabbit anti-β-galactosidase (Millipore). Nuclei were labeled with TO-PRO-3 (Life Technologies). The secondary Abs were Alexa488 (Life Technologies), Cy3 and Cy5 (Jackson ImmunoResearch) donkey IgGs. Images were taken with an LSM510 confocal microscope (Zeiss).

### *In situ* Hybridization

Whole-mount *in situ* hybridization (ISH) in P4 ICR retinas and 10.5 dpc ICR embryos was performed as previously described [36]. The template for *Arhgef15* cRNA probe (1203–2343 of NM_177566) was obtained by reverse transcription of total RNA from P4 ICR mouse retinas with subsequent PCR amplification. After the ISH protocol was completed, the retinal samples were further labeled with rabbit anti-collagen IV polyclonal Ab (Cosmo Bio LSL) and Cy3 anti-rabbit secondary Ab (Jackson ImmunoResearch). Images were taken with an Axioplan2 microscope (Zeiss) equipped with associated software (AxioVision, version 3.1; Zeiss).

### Cell Culture, Transfection, and Immunocytochemistry

HUVECs (Lonza) were grown in Endothelial Growth Medium EGM-2 BulletKit (Lonza). HRECs (Cell Systems) were grown on culture dishes coated with Attachment Factor (Cell Systems) in Complete Medium Kit with Serum and Cultureboost-R (Cell Systems). HUVECs and HRECs were maintained at 37°C in 5% CO_2_, and used for the experiments before passage 7. Plasmid vectors expressing FLAG-tagged human full-length Arhgef15 and its DH-PH truncated domain [15], HA-tagged human WT, constitutively-active (Q79L), and dominant negative (T35N) RhoJ, and FLAG-tagged human constitutively-active Cdc42 (G12V, provided by T. Satoh, Osaka Prefecture University, Osaka, Japan) were transfected using Lipofectamine 2000 and PLUS Reagent (Life Technologies). Stealth RNAi siRNA duplexes (Life Technologies) targeting human Arhgef15 (si-GEF15, HSS117853 and HSS117854) and Cdc42 (si-CDC42, VHS40393), and non-targeting siRNAs (si-Ctrl, Stealth RNAi Negative Control Medium GC Duplex, Life Technologies) were transfected using Lipofectamine RNAiMAX (Life Technologies). At 24 h after plasmid transfection, 72 h after siRNA transfection, or 30 min after stimulation with 500 ng/ml Sema3E protein (R&D Systems), HUVECs were fixed with 4% paraformaldehyde (PFA) in PBS and stained with Alexa Fluor Phalloidin (Life Technologies). Cells transfected with plasmid vectors were detected by co-staining with anti-HA (clone 3F10; Roche Applied Science) or anti-FLAG (clone M2; Sigma-Aldrich) Abs. Images were taken with an Olympus IX81 inverted microscope or an LSM510 confocal microscope.

### Pull-down Assay

HEK-293T cells cultured in Dulbecco’s modified Eagle medium (Sigma-Aldrich) containing 10% FBS were transfected with indicated plasmids with Lipofectamine 2000, and incubated for 48 h before cell lysis. HUVECs pre-treated with siRNA for 72 h were serum-starved with Endothelial Basal Medium EBM-2 (Lonza) containing 1% BSA for 3 h, followed by stimulation with 50 ng/ml human VEGF (HumanZyme) for the indicated periods. The cells were washed with ice-cold PBS, lysed at 4°C in pull-down lysis buffer (20 mM Tris-HCl at pH 7.5, 100 mM NaCl, 10 mM MgCl_2_, 1% TritonX-100, 1 mM ethylene glycol tetraacetic acid, and 1 mM dithiothreitol), and centrifuged for 10 min at 4°C. The supernatants were incubated with GST-PAK-PBD (Cytoskeleton Inc.) conjugated to glutathione agarose beads for 40 min at 4°C, subjected to SDS-PAGE, and transferred onto a polyvinylidene difluoride membrane (Millipore). After blocking with Blocking One (Nacalai Tesque), the membranes were incubated with anti-Cdc42 (clone 44/CDC42; BD Transduction Laboratories) or anti-HA (clone 3F10) Ab, washed, and further incubated with horseradish peroxidase-conjugated anti-mouse (Zymed) or anti-rat (GE healthcare) secondary Abs and ImmunoStar LD (Wako). Aliquots of total cell lysates were also immunoblotted with anti-Cdc42 or anti-HA Ab. Signals were quantified using NIH ImageJ software. The activity of Cdc42 and RhoJ was assessed by calculating the ratio of their GTP-bound forms relative to their total protein amounts. The resulting values were expressed as fold changes compared with the control values (Mock in Figure 2A, si-Ctrl with VEGF 0 min in Figure 2B, and RhoJ-WT in Figure 2C) which were normalized to 1.0. All experiments were repeated three times.

### Cell Proliferation and Apoptosis Assay

At 24 h after siRNA transfection, 1×10^4^ HUVECs were seeded on a 24-well plate and incubated in EGM-2 for 24 h. After serum-starvation with EBM-2 containing 1% BSA for 3 h, cells were incubated with 30 µg/ml 5-bromo-2′-deoxy-uridine (BrdU, Nacalai Tesque) with or without 50 ng/ml human VEGF for 24 h. After fixation with 4% PFA in PBS, cells were labeled with FITC-conjugated anti-BrdU (Roche) or anti-cleaved Caspase-3 (Cell Signaling Technology) Ab. Fluorescence images were taken with an Axio Observer microscope (Zeiss) equipped with associated software (AxioVision, version 4.8.1; Zeiss). The proliferating and apoptotic cells were calculated by averaging the number of BrdU- and Caspase-3-positive cells counted in 2 randomly-selected fields (0.57 mm^2^) per well using WinROOF software (version 6.5, Mitani Corp.). All experiments were repeated three times.

### Scratch-wound Assay

At 24 h after siRNA transfection, 1×10^5^ HUVECs were seeded on a 24-well plate and incubated in EBM-2 containing 1% BSA and 50 ng/ml human VEGF overnight. After the cell monolayers were wounded with 200 µl plastic pipette tips, cells were incubated in EBM-2 containing 1% BSA, 50 ng/ml human VEGF, and 10 µg/ml mitomycin C (Sigma-Aldrich). Images were taken at 0 and 6 h using an Olympus IX-81 inverted microscope. The wound areas were measured using NIH ImageJ software. To quantify wound closure, the wounds at 0 h were considered to be 0% closed.

### Tube Formation Assay

At 48 h after siRNA transfection, 1.5×10^4^ HUVECs were seeded on a 96-well plate pre-coated with the growth factor reduced Matrigel (BD Biosciences). After 10 h incubation in EGM-2, images were taken with an Axio Observer microscope. The total tube length in a fixed area (2.4 mm^2^) of each well was measured using WinROOF software.

### Morphometric Analysis of the Retinal Vasculature

The radius of the retinal vasculature was calculated by averaging the distances from the optic disc to the sprouting vascular fronts in four quadrants per one retina. The vessel area was calculated by measuring the PECAM-1-positive area (except for the optic disc) in whole-mount retina using WinROOF software. The vascular density was assessed by calculating the proportion of the total vessel area to the vascularized retinal area encircled by the sprouting vascular edges. The vessel branch point was calculated by averaging the number of vascular branch points in four fixed retinal areas (1.5×10^5^ µm^2^) per one retina.

### Statistics

Statistical analysis was performed with JMP software (version 8.0.2, SAS Institute Inc.) using an unpaired two-tailed Student’s *t* test, one-way ANOVA with Dunnett’s post-hoc test, or Kruskal-Wallis with Dunn’s post-hoc test. *P* values of less than 0.05 were considered statistically significant.

## Supporting Information

Figure S1 **FACS purification of retinal ECs from P8** ***Tie2GFP*** **Tg mouse.** (A) After removal of dead cells labeled with propidium iodide (PI), doublet cells were eliminated utilizing forward scatter height (FSC-H) versus forward scatter width (FSC-W) gates. (B) Incomplete separation of GFP-positive and GFP-negative cells in retinas of P8 hemizygous *Tie2GFP* Tg mouse.
(TIF)

Figure S2 ***In silico*** **validation of microarray data by the eXintegrator system.** The *x* and *y* axes represent individual samples (*n* = 3 for each population) and the signal intensities, respectively. Red lines indicate borders between sample groupings. Samples are arranged as in Figure 1.
(TIF)

Figure S3 **Expression of the** ***Arhgef15*** **gene in developing vasculature.** (A) Whole-mount ISH for the *Arhgef15* gene and IHC for type IV collagen in P4 mouse retina. Scale bar: 100 µm. (B) Whole-mount ISH for the *Arhgef15* gene in 10.5 dpc mouse embryo.
(TIF)

Figure S4 **Knockdown by siRNA in cultured HUVECs.** Three days after transfection of si-Ctrl, si-CDC42, si-GEF15-1 (HSS117853), and si-GEF15-2 (HSS117854), efficiency of siRNA knockdown was assessed by immunoblotting of cell lysates with anti-human Arhgef15 [15], anti-human Cdc42 (clone 44/CDC42) or anti-β-actin (Sigma-Aldrich) Abs. In experiments presented in Figures 2 and 3, si-GEF15-2 was used.
(TIF)

Figure S5 **Proliferation and apoptosis assays in cultured HUVECs.** (A and B) Quantification of BrdU-positive (A) and Caspase-3-positive (B) cells in the presence or absence of VEGF (*n* = 3 per group). Error bars represent SEM; **P<0.01.
(TIF)

Figure S6 **Arhgef15 facilitates actin polymerization in retinal ECs.** Confocal microscopy for phalloidin in cultured HRECs after siRNA transfection or Sema3E stimulation. Scale bar: 10 µm.
(TIF)

Figure S7 **A scheme for generating** ***Arhgef15*** **-KO mouse.** TT2 ES cells were electroporated with the linearized targeting construct, selected with G418, and screened by PCR. Positive clones were further confirmed by Southern blot using a 500 bp probe outside the 3′ arm (EcoRI digest), generating an 11 kb WT and a 4 kb targeted band. Out of 7 ES cell clones carrying the correct mutation, the #19 clone was injected into ICR embryos. E, EcoRI; As, Asp718; RV, EcoRV.
(TIF)

Figure S8 **Absence of Arhgef15 expression in non-ECs of P5** ***Arhgef15*** **^lacZ/lacZ^ retinas.** (A) In sprouting vessels, Arhgef15 was expressed both in tip (arrows) and stalk ECs (arrowheads). (B) In retinal cryo-sections, Arhgef15 expression was detected in vascular ECs, but not in neural and glial cells. (C and D) In retinal blood vessels, Arhgef15 expression was undetectable in PDGFRα-positive astrocytes (C) and αSMA-positive vSMCs. Scale bar: 10 µm (A, C, and D); 20 µm (B).
(TIF)

Figure S9 **Formation of the superficial and deeper vascular layers in** ***Arhgef15*** **-KO retinas.** (A and B) Whole-mount IHC for PECAM-1 in P10 (A) and P13 (B) retinas. In (A), the growing blood vessels reached the retinal periphery (arrow) of *Arhgef15-KO* mice. Scale bar: 200 µm (A); 50 µm (B).
(TIF)

Table S1 **List of 56 endothelial genes encoding G proteins.**
(DOC)

Table S2 **Gene ontology analysis of retinal endothelial genes.**
(DOC)

Dataset S1 **List of 1,623 probe sets upregulated in ECs in P8 mouse retinas.**
(XLS)

Dataset S2 **List of 96 probe sets for endothelial G proteins.**
(XLS)
