# Supplementary figures and images for: Arhgef15 Promotes Retinal Angiogenesis by Mediating VEGF-Induced Cdc42 Activation and Potentiating RhoJ Inactivation in Endothelial Cells
Source: PLoS One. 2012 Sep 21;7(9):e45858. doi: 10.1371/journal.pone.0045858 (PMC3448698; doi:10.1371/journal.pone.0045858)

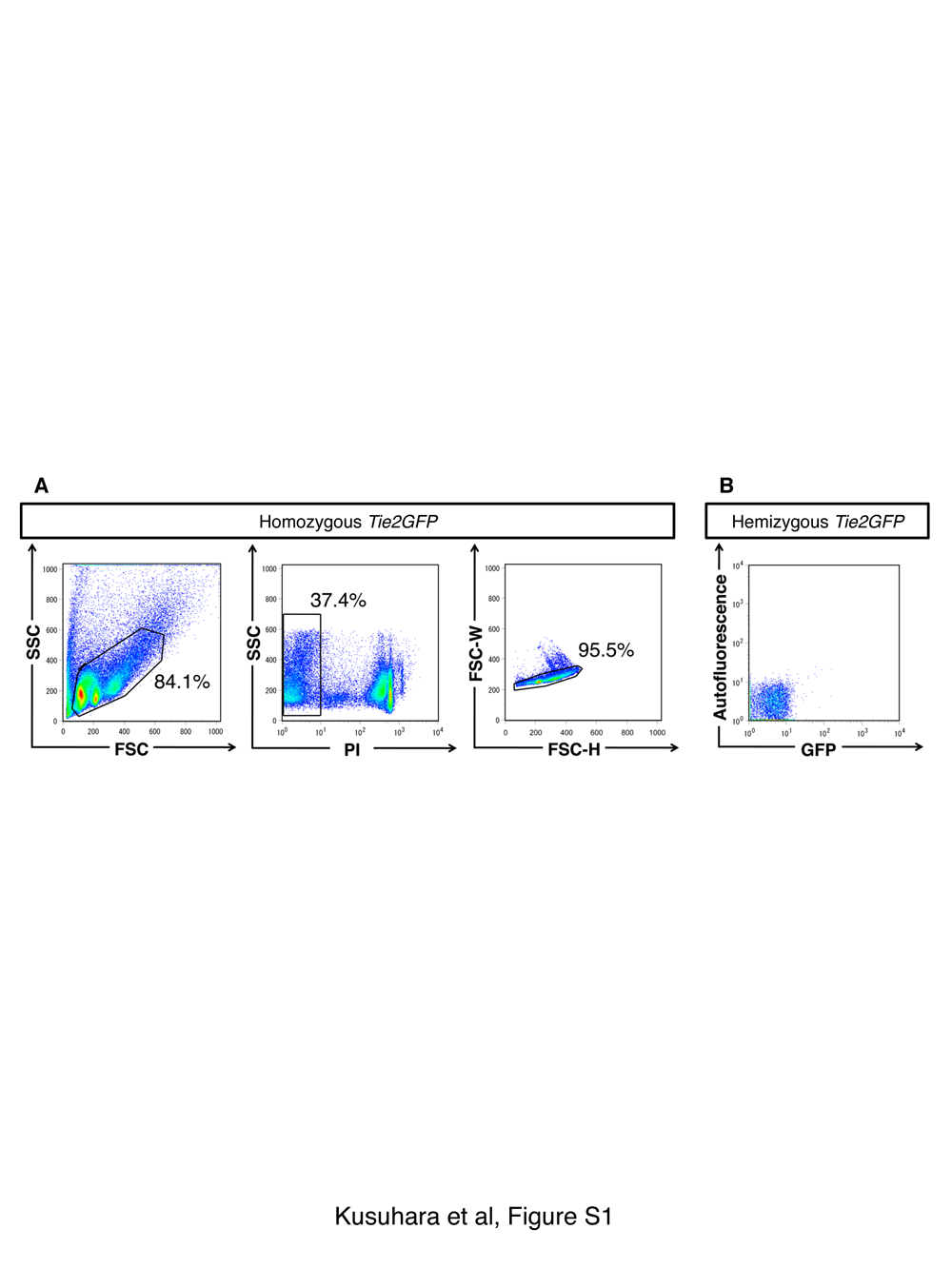

Supplement: Figure S1 — FACS purification of retinal ECs from P8 Tie2GFP Tg mouse. (A) After removal of dead cells labeled with propidium iodide (PI), doublet cells were eliminated utilizing forward scatter height (FSC-H) versus forward scatter width (FSC-W) gates. (B) Incomplete separation of GFP-positive and GFP-negative cells in retinas of P8 hemizygous Tie2GFP Tg mouse. (TIF) [file pone.0045858.s001.tif]

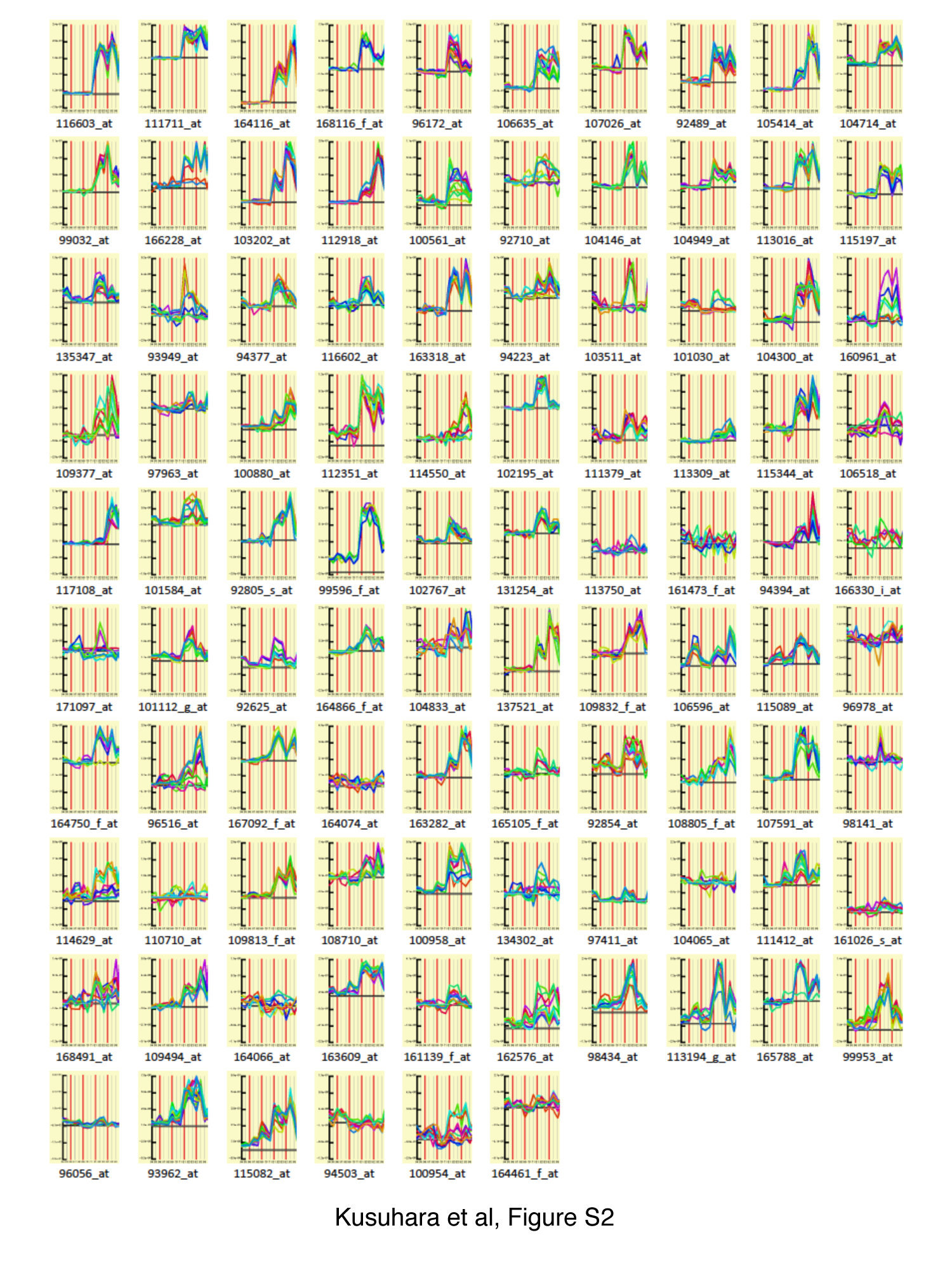

Supplement: Figure S2 — In silico validation of microarray data by the eXintegrator system. The x and y axes represent individual samples (n = 3 for each population) and the signal intensities, respectively. Red lines indicate borders between sample groupings. Samples are arranged as in Figure 1. (TIF) [file pone.0045858.s002.tif]

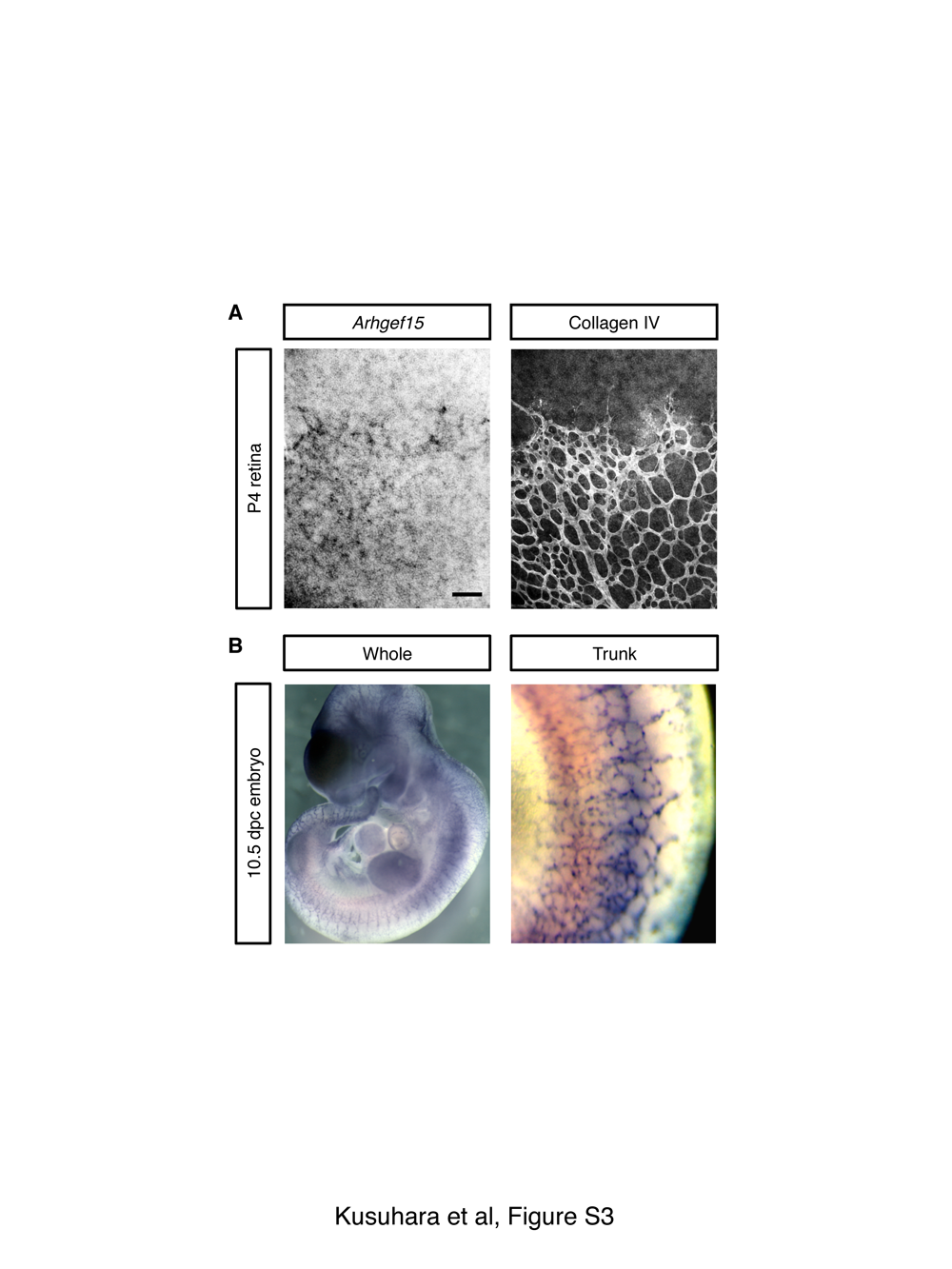

Supplement: Figure S3 — Expression of the Arhgef15 gene in developing vasculature. (A) Whole-mount ISH for the Arhgef15 gene and IHC for type IV collagen in P4 mouse retina. Scale bar: 100 µm. (B) Whole-mount ISH for the Arhgef15 gene in 10.5 dpc mouse embryo. (TIF) [file pone.0045858.s003.tif]

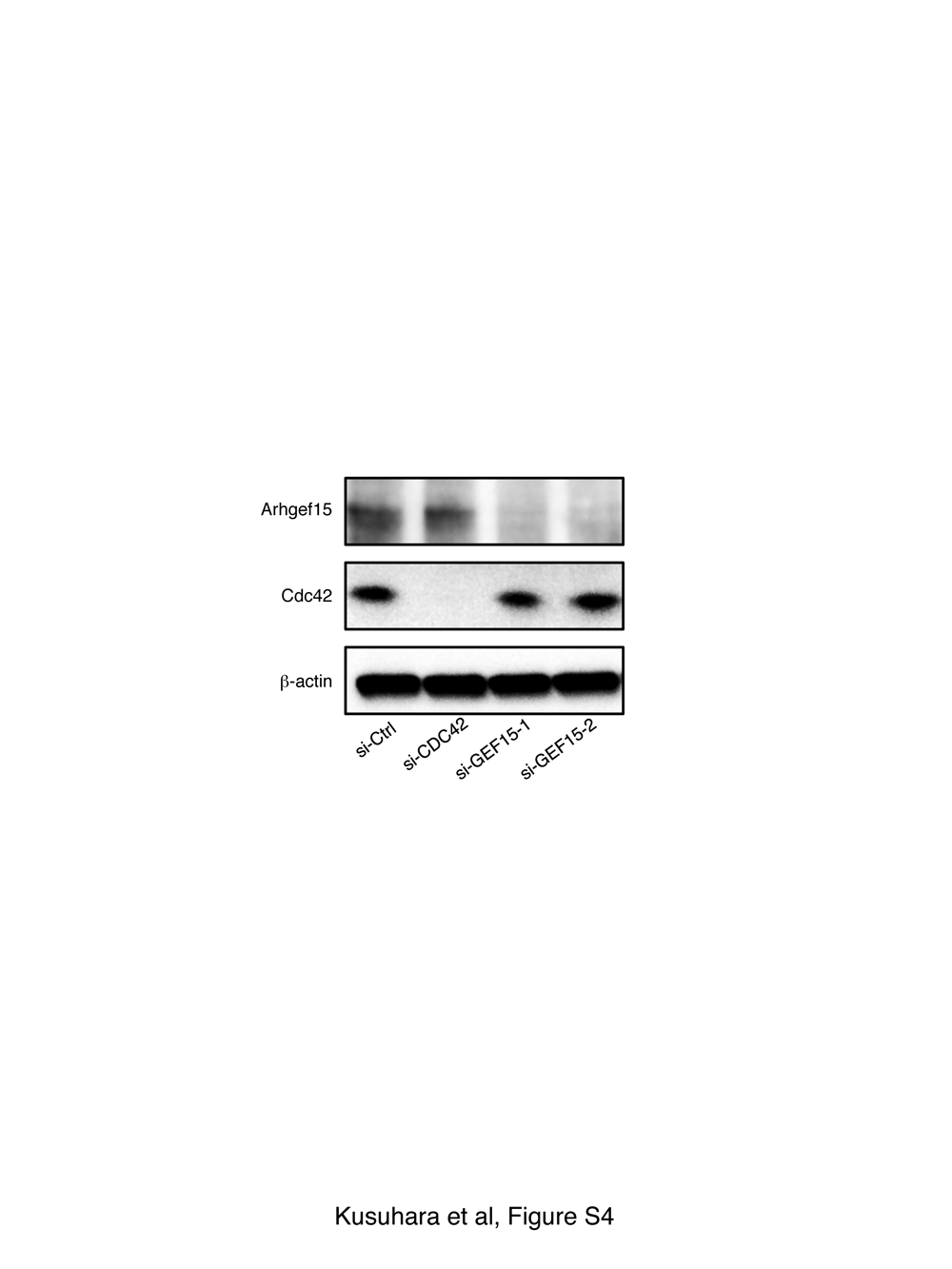

Supplement: Figure S4 — Knockdown by siRNA in cultured HUVECs. Three days after transfection of si-Ctrl, si-CDC42, si-GEF15-1 (HSS117853), and si-GEF15-2 (HSS117854), efficiency of siRNA knockdown was assessed by immunoblotting of cell lysates with anti-human Arhgef15 [15], anti-human Cdc42 (clone 44/CDC42) or anti-β-actin (Sigma-Aldrich) Abs. In experiments presented in Figures 2 and 3, si-GEF15-2 was used. (TIF) [file pone.0045858.s004.tif]

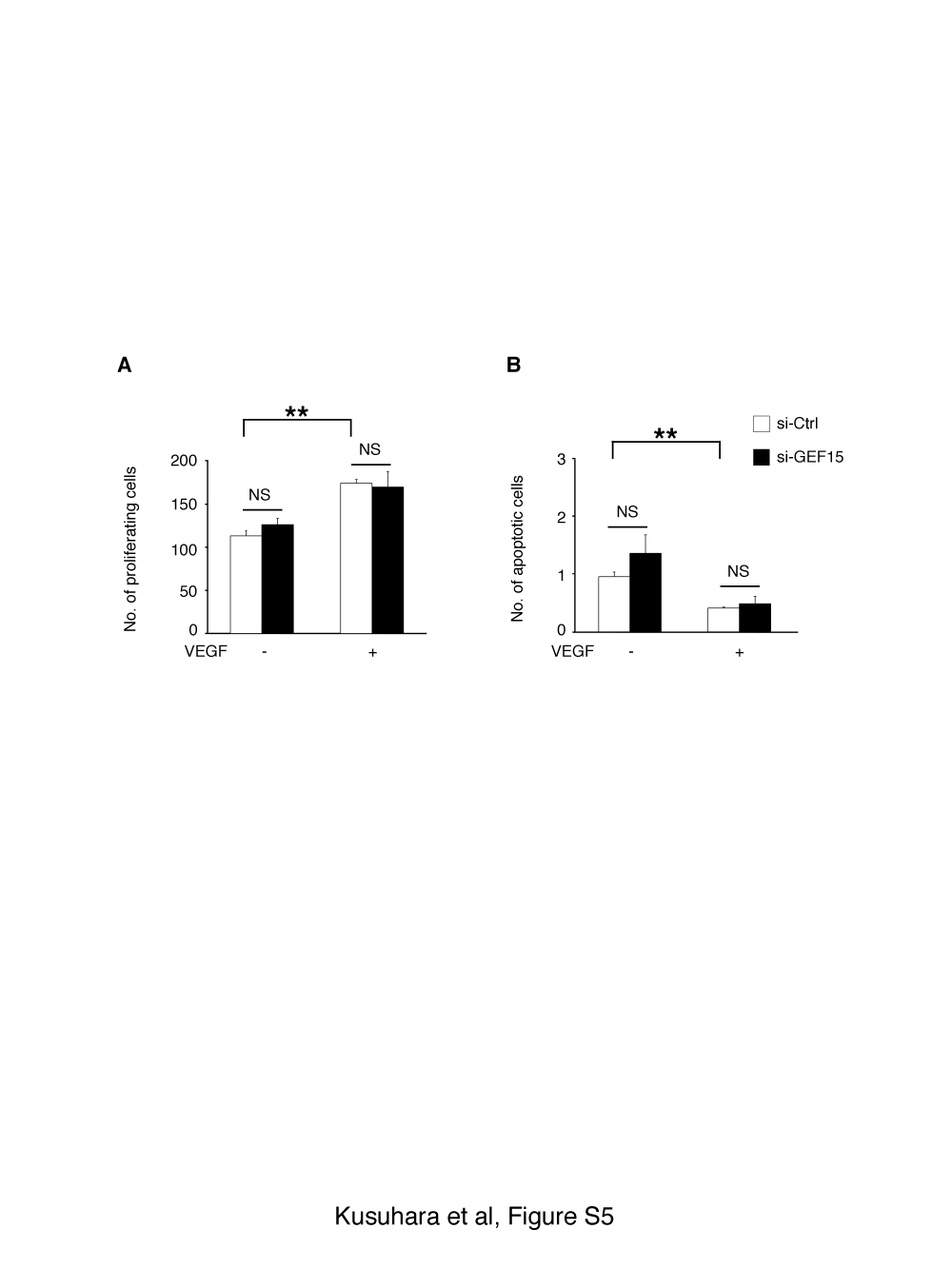

Supplement: Figure S5 — Proliferation and apoptosis assays in cultured HUVECs. (A and B) Quantification of BrdU-positive (A) and Caspase-3-positive (B) cells in the presence or absence of VEGF (n = 3 per group). Error bars represent SEM; **P<0.01. (TIF) [file pone.0045858.s005.tif]

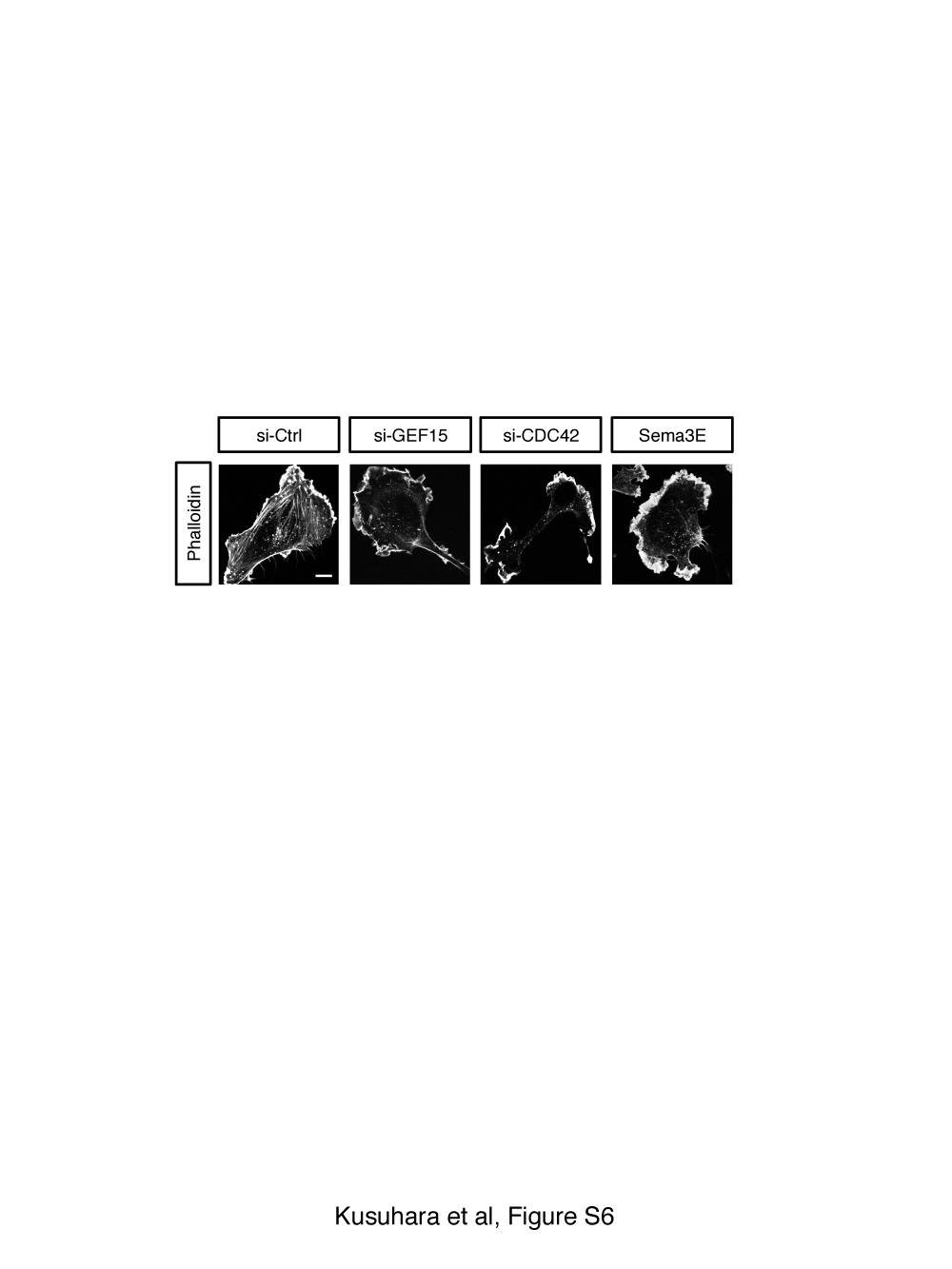

Supplement: Figure S6 — Arhgef15 facilitates actin polymerization in retinal ECs. Confocal microscopy for phalloidin in cultured HRECs after siRNA transfection or Sema3E stimulation. Scale bar: 10 µm. (TIF) [file pone.0045858.s006.tif]

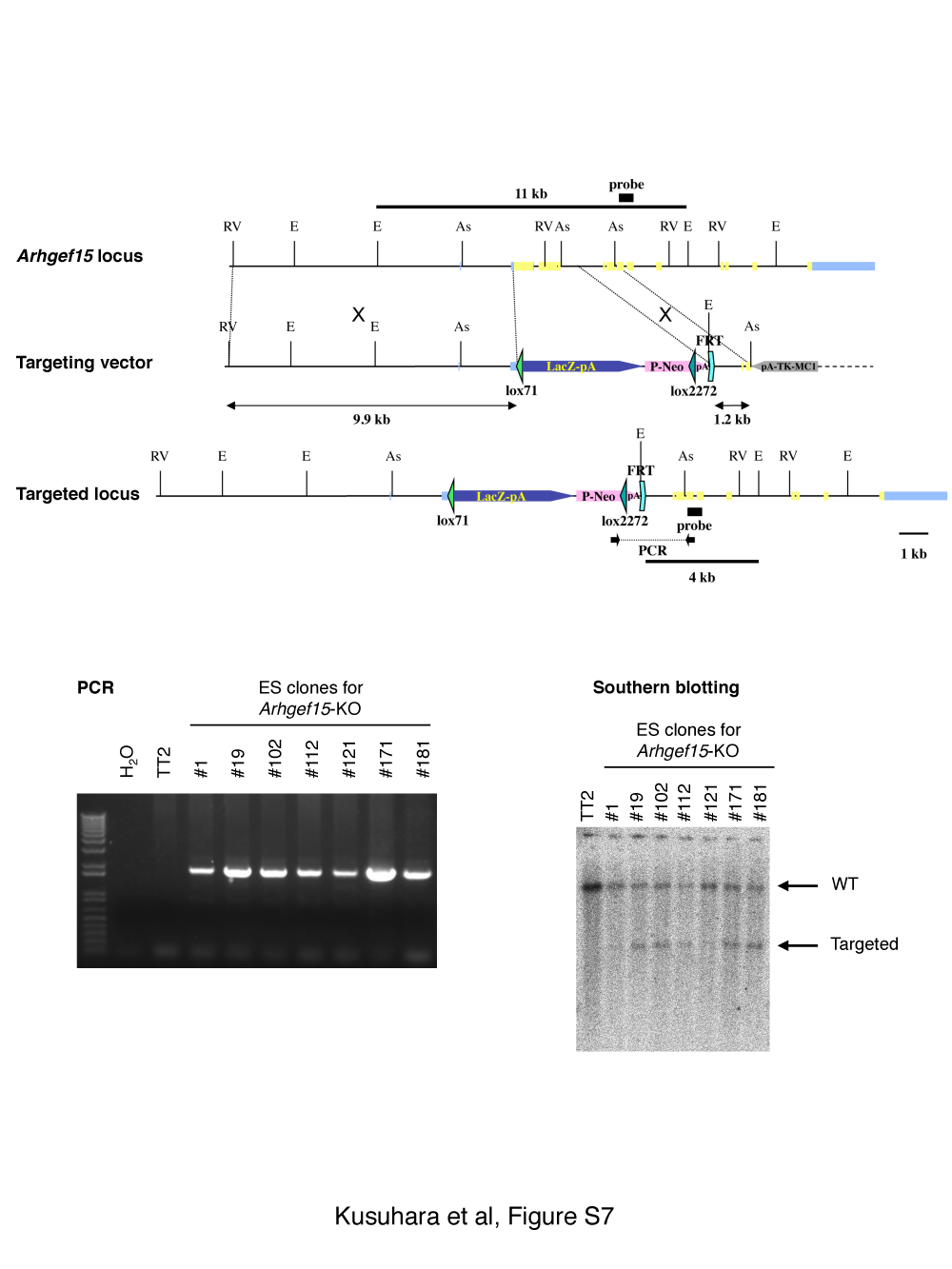

Supplement: Figure S7 — A scheme for generating Arhgef15 -KO mouse. TT2 ES cells were electroporated with the linearized targeting construct, selected with G418, and screened by PCR. Positive clones were further confirmed by Southern blot using a 500 bp probe outside the 3′ arm (EcoRI digest), generating an 11 kb WT and a 4 kb targeted band. Out of 7 ES cell clones carrying the correct mutation, the #19 clone was injected into ICR embryos. E, EcoRI; As, Asp718; RV, EcoRV. (TIF) [file pone.0045858.s007.tif]

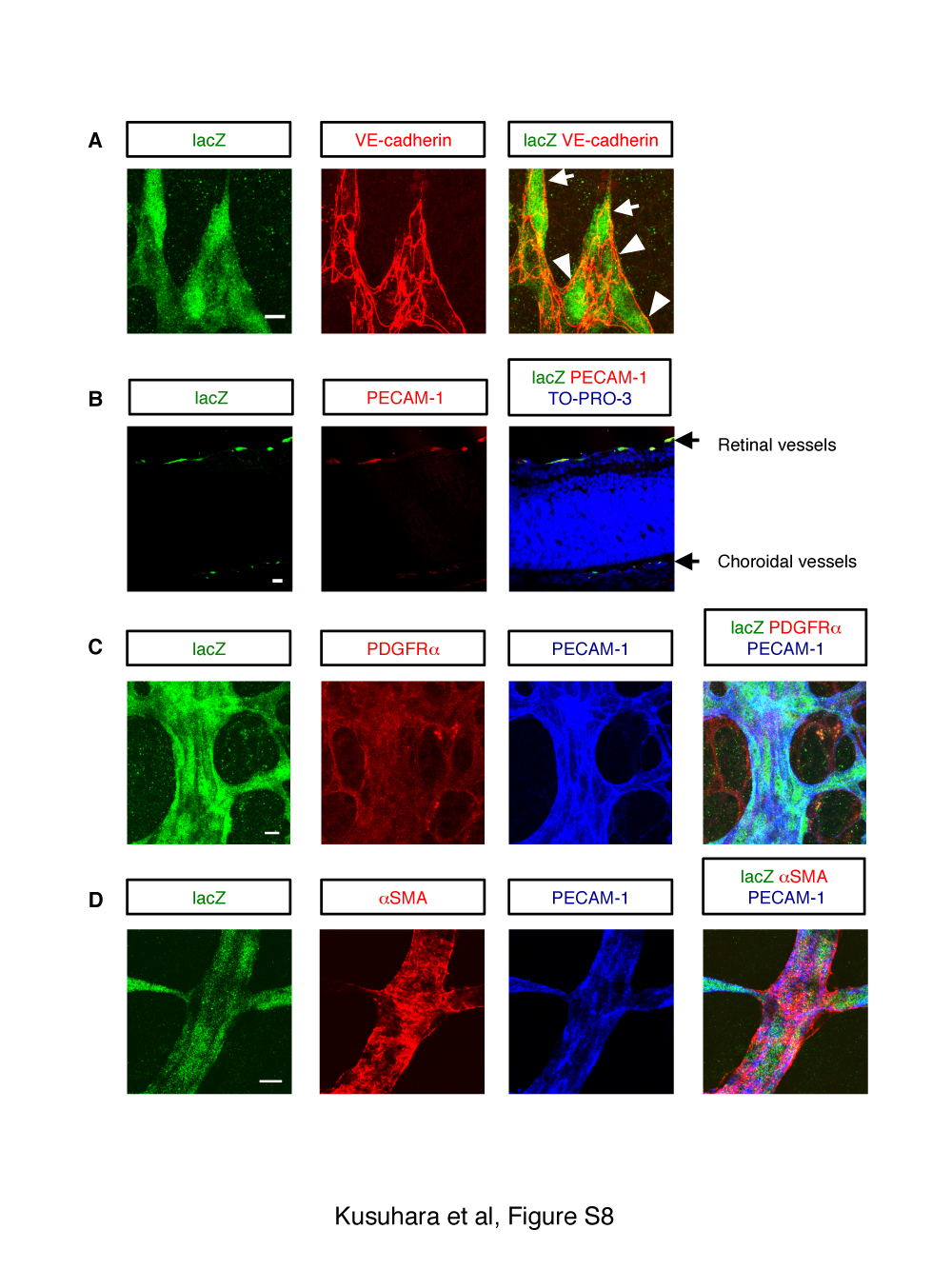

Supplement: Figure S8 — Absence of Arhgef15 expression in non-ECs of P5 Arhgef15 lacZ/lacZ retinas. (A) In sprouting vessels, Arhgef15 was expressed both in tip (arrows) and stalk ECs (arrowheads). (B) In retinal cryo-sections, Arhgef15 expression was detected in vascular ECs, but not in neural and glial cells. (C and D) In retinal blood vessels, Arhgef15 expression was undetectable in PDGFRα-positive astrocytes (C) and αSMA-positive vSMCs. Scale bar: 10 µm (A, C, and D); 20 µm (B). (TIF) [file pone.0045858.s008.tif]

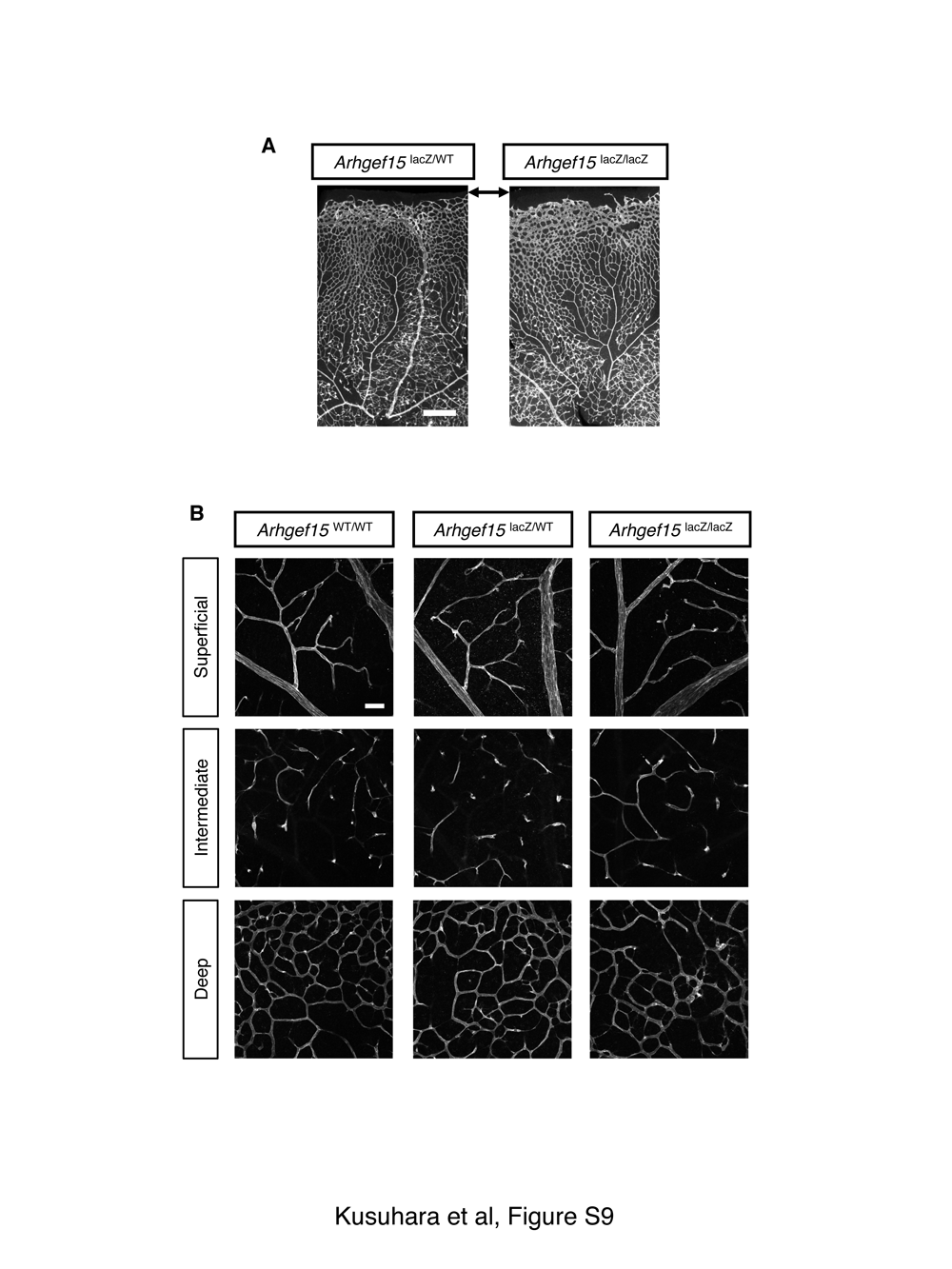

Supplement: Figure S9 — Formation of the superficial and deeper vascular layers in Arhgef15 -KO retinas. (A and B) Whole-mount IHC for PECAM-1 in P10 (A) and P13 (B) retinas. In (A), the growing blood vessels reached the retinal periphery (arrow) of Arhgef15-KO mice. Scale bar: 200 µm (A); 50 µm (B). (TIF) [file pone.0045858.s009.tif]
