# Supplementary material for: Arhgef15 Promotes Retinal Angiogenesis by Mediating VEGF-Induced Cdc42 Activation and Potentiating RhoJ Inactivation in Endothelial Cells
Source: PLoS One. 2012 Sep 21;7(9):e45858. doi: 10.1371/journal.pone.0045858 (PMC3448698; doi:10.1371/journal.pone.0045858)
Supplement: Table S1 — List of 56 endothelial genes encoding G proteins. (DOC) [file pone.0045858.s010.doc]

**Table S1. List of 56 endothelial genes encoding G proteins.**

**No. Symbol Name Probe set Fold**

1 *Rasgrp3* RAS, guanyl releasing protein3 116603_at 67.02

116602_at* 6.18

2 *Gimap8* GTPase, IMAP family member 8 111711_at 26.38

3 *Fgd5* FYVE, RhoGEF and PH domain containing 5 164116_at 21.28

4 *Arhgap25* Rho GTPase activating protein 25 168116_f_at 18.79

5 *Gimap4* GTPase, IMAP family member 4 96172_at 18.62

6 *Gimap6* GTPase, IMAP family member 6 106635_at 12.93

7 *Gnb4* guanine nucleotide binding protein (G protein), beta 4 107026_at 12.78

8 *Gimap1* GTPase, IMAP family member 1 92489_at 11.94

9 *Gch1* GTP cyclohydrolase 1 105414_at 11.74

10 *Dock9* dedicator of cytokinesis 9 104714_at 11.6

11 *Rasd1* RAS, dexamethasone-induced 1 99032_at 11.53

12 *Gbp4* guanylate binding protein 4 103202_at 10.16

13 *Centd1* centaurin, delta 1 (Arap2) 112918_at 9.73

14 *Iqgap1* IQ motif containing GTPase activating protein 1 100561_at 9.50

104300_at* 5.00

15 *Rasip1* Ras interacting protein 1 104146_at 9.14

16 *Arhgef15* Rho guanine nucleotide exchange factor (GEF) 15 113016_at 8.49

17 *LOC670024* similar to Dedicator of cytokinesis protein 6 115197_at 7.44

18 *Gng11* guanine nucleotide binding protein (G protein), gamma 11 94377_at 6.27

19 *Rapgef4* Rap guanine nucleotide exchange factor (GEF) 4 163318_at 5.61

20 *Net1* neuroepithelial cell transforming gene 1 94223_at 5.43

21 *Rapgef3* Rap guanine nucleotide exchange factor (GEF) 3 109377_at 4.29

22 *Sipa1* signal-induced proliferation associated gene 1 97963_at 4.20

23 *Gbp7* guanylate binding protein 7 100880_at 4.06

24 *Rapgef6* Rap guanine nucleotide exchange factor (GEF) 6 112351_at 3.89

25 *Map4k4* mitogen-activated protein kinase kinase kinase kinase 4 102195_at 3.77

26 *Stard8* START domain containing 8 113309_at 3.59

27 *Rgs3* regulator of G-protein signaling 3 115344_at 3.56

28 *Mpa2l* macrophage activation 2 like (Gbp6) 117108_at 3.53

29 *Rsu1* Ras suppressor protein 1 101584_at 3.49

30 *Arl4a* ADP-ribosylation factor-like 4A 92805_s_at 3.48

108805_f_at* 2.66

31 *Gnai2* guanine nucleotide binding protein (G protein), alpha inhibiting 2 99596_f_at 3.46

32 *Gng12* guanine nucleotide binding protein (G protein), gamma 12 102767_at 3.31

33 *Rras* Harvey rat sarcoma oncogene, subgroup R 94394_at 3.21

34 *Rhoa* ras homolog gene family, member A 101112_g_at 3.10

35 *Nme2* non-metastatic cells 2, protein (NM23B) expressed in 92625_at 3.09

36 *Srgap2* SLIT-ROBO Rho GTPase activating protein 2 104833_at 3.04

37 *-* - 137521_at 3.03

38 *-* - 109832_f_at 3.01

39 *Cdgap* Cdc42 GTPase-activating protein (Arhgap31) 106596_at 2.96

40 *Rapgef5* Rap guanine nucleotide exchange factor (GEF) 5 115089_at 2.92

41 *1810048P08Rik* RIKEN cDNA 1810048P08 gene (Rab43) 167092_f_at 2.83

42 *Rhobtb1* Rho-related BTB domain containing 1 163282_at 2.78

43 *Rab11a* RAB11a, member RAS oncogene family 92854_at 2.68

44 *Rhoj* ras homolog gene family, member J 107591_at 2.65

45 *Eif5b* eukaryotic translation initiation factor 5B 98141_at 2.63

46 *Git2* G protein-coupled receptor kinase-interactor 2 109813_f_at 2.56

47 *Spata13* spermatogenesis associated 13 100958_at 2.50

48 *Setd6* SET domain containing 6 111412_at 2.43

49 *Dock1* dedicator of cytokinesis 1 109494_at 2.32

163609_at* 2.22

50 *Rras2* related RAS viral (r-ras) oncogene homolog 2 162576_at 2.21

51 *Arhgef7* Rho guanine nucleotide exchange factor (GEF) 7 98434_at 2.18

52 *Rapgef1* Rap guanine nucleotide exchange factor (GEF) 1 113194_g_at 2.15

53 *Rgl2* ral guanine nucleotide dissociation stimulator-like 2 99953_at 2.08

54 *Rhoc* ras homolog gene family, member C 96056_at 2.06

55 *Rap1a* RAS-related protein-1a 93962_at 2.06

56 *Arhgef12* Rho guanine nucleotide exchange factor (GEF) 12 115082_at 2.03

* indicates probe sets for overlapping genes.
