# Supplementary material for: Arhgef15 Promotes Retinal Angiogenesis by Mediating VEGF-Induced Cdc42 Activation and Potentiating RhoJ Inactivation in Endothelial Cells
Source: PLoS One. 2012 Sep 21;7(9):e45858. doi: 10.1371/journal.pone.0045858 (PMC3448698; doi:10.1371/journal.pone.0045858)
Supplement: Table S2 — Gene ontology analysis of retinal endothelial genes. (DOC) [file pone.0045858.s011.doc]

**Table S2. Gene ontology analysis of retinal endothelial genes.**

**Biological process (level 3) Count % *P***

regulation of cell motility 27 1.87 7.91E-14

regulation of locomotion 27 1.87 3.45E-13

cell motility 59 4.09 5.36E-11

angiogenesis 34 2.36 2.92E-10

system development 172 11.93 1.61E-09

cell migration 49 3.40 2.77E-09

negative regulation of biological process 114 7.91 3.05E-09

regulation of cellular process 318 22.05 6.26E-09

negative regulation of cellular process 106 7.35 1.76E-08

anatomical structure formation 37 2.57 1.97E-08

mitotic cell cycle 42 2.91 5.02E-08

organ development 140 9.71 5.83E-08

positive regulation of biological process 109 7.56 6.20E-08

anatomical structure morphogenesis 122 8.46 7.61E-08

positive regulation of cellular process 98 6.80 9.47E-08

cell cycle process 72 4.99 1.57E-07

cell cycle phase 45 3.12 2.53E-07

organ morphogenesis 64 4.44 8.08E-07

regulation of cell proliferation 50 3.47 9.23E-07

cell differentiation 167 11.58 9.43E-07

regulation of signal transduction 56 3.88 1.73E-06

embryonic development 56 3.88 2.97E-06

regulation of angiogenesis 12 0.83 8.45E-06

positive regulation of locomotion 9 0.62 2.40E-05

negative regulation of locomotion 10 0.69 2.58E-05

organelle organization and biogenesis 114 7.91 3.90E-05

regulation of cell cycle 45 3.12 5.03E-05
